# Supplementary material for: Decreased clot burden is associated with factor XIII Val34Leu polymorphism and better functional outcomes in acute ischemic stroke patients treated with intravenous thrombolysis
Source: PLoS One. 2021 Jul 7;16(7):e0254253. doi: 10.1371/journal.pone.0254253 (PMC8263307; doi:10.1371/journal.pone.0254253)
Supplement: S1 Table — (DOCX) [file pone.0254253.s001.docx]

**S1 Table. Effects of fibrinogen and FXIII-A Val34 or Leu34 alleles on clot burden score.**

|  | **FXIII^Val/Val^** | | | **FXIII^Val/Leu^** | | | **FXIII^Leu/Leu^** | | |  |
| --- | --- | --- | --- | --- | --- | --- | --- | --- | --- | --- |
|  | **fibrinogen < 3.5 g/L**  **(N=32)** | **fibrinogen > 3.5 g/L**  **(N=77)** | ***P*** | **fibrinogen**  **< 3.5 g/L**  **(N=25)** | **fibrinogen**  **> 3.5 g/L**  **(N=51)** | ***P*** | **fibrinogen < 3.5 g/L**  **(N=2)** | **fibrinogen > 3.5 g/L**  **(N=8)** | ***P*** | ***P***^*^ |
| Clot burden score | 9 (7.5-10) | 9 (7-10) | 0.4228 | 10 (8-10) | 10 (6-10) | 0.4155 | 10 (10-10) | 10 (8.5-10) | 0.3363 | 0.4427 |

Data show median and interquartile range. Groups were compared using Kruskal Wallis with Dunn’s post-hoc test. ^*^Kruskal Wallis comparing all groups
