# Supplementary material for: Decreased clot burden is associated with factor XIII Val34Leu polymorphism and better functional outcomes in acute ischemic stroke patients treated with intravenous thrombolysis
Source: PLoS One. 2021 Jul 7;16(7):e0254253. doi: 10.1371/journal.pone.0254253 (PMC8263307; doi:10.1371/journal.pone.0254253)
Supplement: S2 Table — (DOCX) [file pone.0254253.s002.docx]

**S2 Table. Levels of different hemostasis markers according to CBS, median (IQR)**

|  | CBS 0-5  (n=28) | CBS 6-7  (n=29) | CBS 8-9  (n=43) | CBS 10  n=100 | P |
| --- | --- | --- | --- | --- | --- |
| Plasminogen activity on admission (%) | 114.00 (92.00-133.00) | 105.00 (94.00-128.00) | 114.00 (90.00-134.00) | 97.00 (88.00-118.00) | 0.153 |
| Plasminogen activity at 24 h (%) | 100.50 (82.50-114.50) | 81.00 (73.00-105.00) | 90.50 (78.00-104.00) | 89.00 (81.00-101.00) | 0.069 |
| α2-plasmin inhibitor activity on admission (%) | 104.00 (98.50-112.00) | 97.50 (89.00-106.50) | 100.00 (92.50-109.00) | 101.00 (89.00-109.00) | 0.186 |
| α2-plasmin inhibitor activity at 24 h (%) | 76.50 (69.00-89.00) | 76.00 (69.00-86.00) | 71.50 (63.00-80.00) | 76.00 (67.00-86.00) | 0.222 |
| D-dimer level on admission (mg/L) | 0.71 (0.53-1.33) | 0.89 (0.65-1.62) | 0.83 (0.45-1.55) | 0.70 (0.51-1.60) | 0.450 |
| D-dimer level at 24 h (mg/L) | 2.60 (1.17-3.74) | 2.69 (1.37-3.96) | 2.50 (1.41-7.19) | 1.85 (1.03-5.17) | 0.237 |
| Fibrinogen level on admission (g/L) | 3.97 (3.50-4.50) | 4.22 (3.46-4.77) | 3.75 (3.46-4.49) | 3.91 (3.21-4.54) | 0.535 |
| Fibrinogen at 24 h (g/L) | 3.68 (2.83-4.13) | 4.01 (3.46-4.70) | 3.58 (2.93-4.14) | 3.76 (3.18-4.31) | 0.293 |
| Factor XIII activity on admission (%) | 128.00 (70.32-146.23) | 117.41 (89.49-134.59) | 111.25 (93.05-141.9) | 131.27 (95.22-165.84) | 0.138 |
| Factor XIII activity at 24 h (%) | 119.79 (95.99- 130.50) | 115.20 (85.85-125.76) | 109.00 (85.97- 134.83) | 104.26 (80.61- 130.33) | 0.508 |

CBS, clot burden score; IQR, interquartile range
